# Functionalized Lipid Nanocarriers for Simultaneous Delivery of Docetaxel and Tariquidar to Chemoresistant Cancer Cells

**DOI:** 10.3390/ph16030349

**Published:** 2023-02-24

**Authors:** Chang Hyun Kim, Sangkil Lee, Ji Yeh Choi, Min Jeong Lyu, Hyun Min Jung, Yoon Tae Goo, Myung Joo Kang, Young Wook Choi

**Affiliations:** 1 College of Pharmacy, Chung-Ang University, 84 Heukseok-ro, Dongjak-gu, Seoul 06974, Republic of Korea; 2 College of Pharmacy, Keimyung University, 1095 Dalgubeol-daero, Dalseo-gu, Daegu 42601, Republic of Korea; 3 Department of Psychology, York University, 4700 Kneele St., Toronto, ON M3J 1P3, Canada; 4 College of Pharmacy, Dankook University, 119 Dandae-ro, Dongnam-gu, Cheonan 31116, Republic of Korea

**Keywords:** multidrug resistance, P-gp inhibitor, docetaxel, tariquidar, co-delivery, nanostructured lipid carrier

## Abstract

The simultaneous drug delivery efficiency of a co-loaded single-carrier system of docetaxel (DTX)- and tariquidar (TRQ)-loaded nanostructured lipid carrier (NLC) functionalized with PEG and RIPL peptide (PRN) (D^T-PRN) was compared with that of a physically mixed dual-carrier system of DTX-loaded PRN (D-PRN) and TRQ-loaded PRN (T-PRN) to overcome DTX mono-administration-induced multidrug resistance. NLC samples were prepared using the solvent emulsification evaporation technique and showed homogeneous spherical morphology, with nano-sized dispersion (<220 nm) and zeta potential values of −15 to −7 mV. DTX and/or TRQ was successfully encapsulated in NLC samples (>95% encapsulation efficiency and 73–78 µg/mg drug loading). In vitro cytotoxicity was concentration-dependent; D^T-PRN exhibited the highest MDR reversal efficiency, with the lowest combination index value, and increased the cytotoxicity and apoptosis in MCF7/ADR cells by inducing cell-cycle arrest in the G2/M phase. A competitive cellular uptake assay using fluorescent probes showed that, compared to the dual nanocarrier system, the single nanocarrier system exhibited better intracellular delivery efficiency of multiple probes to target cells. In the MCF7/ADR-xenografted mouse models, simultaneous DTX and TRQ delivery using D^T-PRN significantly suppressed tumor growth as compared to other treatments. A single co-loaded system for PRN-based co-delivery of DTX/TRQ (1:1, *w*/*w*) constitutes a promising therapeutic strategy for drug-resistant breast cancer cells.

## 1. Introduction

Docetaxel (DTX) is a second-generation taxane that disrupts microtubule stabilization to induce mitotic arrest and apoptosis [1]. DTX is clinically used to treat solid tumors, such as breast, ovarian, prostate, and non-small cell lung cancers [2,3], albeit with limited applicability owing to the low aqueous solubility, nonspecific in vivo biodistribution, and the development of multidrug resistance (MDR) in cancer cells [4,5]. The overexpression of the transmembrane drug efflux transporter P-glycoprotein (P-gp) is a key mechanism of MDR [6,7], and the P-gp transporter mediates cellular drug resistance through the efflux of a broad range of chemotherapeutic agents, including taxanes, anthracyclines, and vinca alkaloids [8]. Consequently, cytoplasmic DTX accumulation is diminished by drug efflux, with the resultant low efficacy and bioavailability in MDR cancer [4].

Delivery combinations of P-gp inhibitors and DTX have been studied extensively to improve therapeutic outcomes in MDR cancers [9]. Tariquidar (TRQ) is a third-generation P-gp inhibitor that is more potent and specific to P-gp and has fewer interactions with the cytochrome P450 3A4 system as compared with the first- and second-generation P-gp inhibitors [10]. TRQ is a non-competitive P-gp inhibitor that hinders adenosine triphosphate binding/hydrolysis without inducing conformational changes in P-gp [11]. Although TRQ may improve the therapeutic efficacy of anticancer drugs against drug-resistant cancer when administered in combination, the poor aqueous solubility, adverse effects induced by TRQ exposure of normal cells, and nonspecific distribution and nonuniform action within the body limit the clinical application of TRQ [12,13]. Moreover, TRQ should be co-delivered to the same target cells as the chemotherapeutic agent to achieve synergistic anticancer activity [14].

Targeted nanoparticulate carriers constitute novel platforms for enhancing MDR reversal in combination therapy [9,14], and among them, nanostructured lipid carriers (NLCs) have been widely applied due to their high hydrophobic drug-loading capacity, excellent biocompatible property, and ease of surface functionalization for targeting and prolonging the in vivo circulation of chemotherapeutic drugs [15,16]. Guo et al. reported cetuximab-functionalized, paclitaxel, and 5-demethylnobiletin co-loaded NLCs for synergistic combination therapy of lung cancer [17]. Zhang et al. co-encapsulated paclitaxel and chlorin e6 on the folic acid-modified NLC to treat breast cancer [18]. We identified a novel cell-penetrating homing peptide—the RIPL peptide (IPLVVPLRRRRRRRRC) [19,20]—and developed a novel hepsin (Hpn)-specific targeted delivery system that utilizes PEGylated (5 mol%) and RIPL peptide-conjugated NLC (PRN) [21,22]. Previous studies demonstrated that the specificity to target cancer cells and the enhanced internalization of drug molecules into the cytoplasm were achieved by functionalized NLC systems that use the RIPL peptide [22,23]. Thus, as a targeted drug delivery platform, PRN has been applied in the simultaneous delivery of drug combinations.

Co-delivery of multiple drugs using a nanocarrier system necessitates either drug loading into separate nanoparticles (dual-type) or co-encapsulation into a single nanoparticle (single-type). Co-administration of curcumin or TRQ with DTX using PRN as a dual-type nanocarrier system increased the in vitro efficacy of DTX in drug-resistant MCF7/ADR breast cancer cells [24,25]. The dual-type nanocarrier system is obtained by physically mixing two separate drug-loaded nanocarriers and has the following advantages: encapsulating drugs that have different physical properties, controlling the drug-loading amounts, or providing additional functionality as needed [26]. Moreover, target specificity and enhanced internalization of drug molecules can be achieved with the nanocarrier system. Although dual-type nanocarriers offer flexibility in dosing and prevent drug–drug interactions, an optimal synergistic effect may not be attained because of different cellular uptake characteristics of separate nanocarriers [26,27]. In contrast, co-loaded single-carrier drug-delivery systems can achieve multidrug spatial and temporal delivery by synchronizing biodistribution and intra-tumoral drug accumulation with an optimized combination ratio [26,28]. Furthermore, considering the mechanism of action of DTX and TRQ, it was expected that co-localization into the same target cells using a single-type nanocarrier system could significantly increase the anticancer efficacy. Thus, it was necessary to design a single-type NLC that can encapsulate both DTX and TRQ while maintaining the optimized drug–drug ratio to achieve more advanced, precise, simultaneous multidrug delivery.

**Figure 1:**
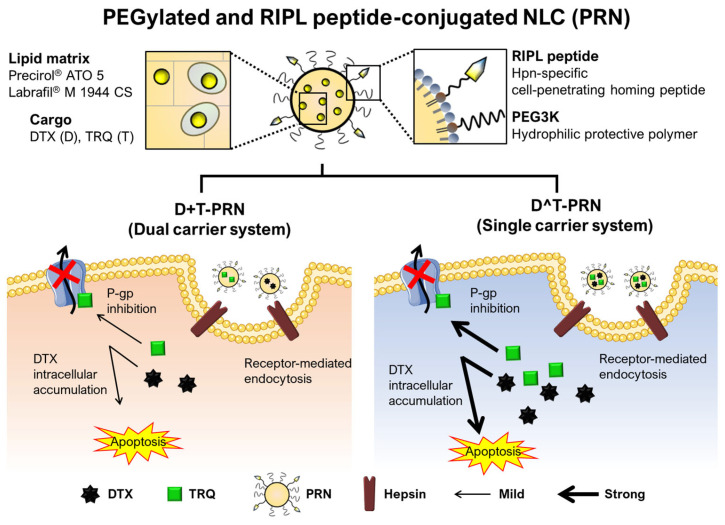
Schematic illustration of the co-delivery system and the proposed cell apoptosis pathway induced by the docetaxel (DTX)- and/or tariquidar (TRQ)-loaded PEGylated (5 mol%) and RIPL peptide-conjugated nanostructured lipid carrier (PRN). Hydrophobic cargos (DTX and TRQ) are loaded separately into different PRNs (dual-carrier system) or simultaneously co-loaded into a PRN (single-carrier system). The lipid matrix of the PRN is composed of both solid lipid (Precirol^®^ ATO 5) and liquid oil (Labrafil^®^ M 1944 CS). The PRN surface is functionalized with a hepsin (Hpn)-specific cell-penetrating homing peptide (RIPL peptide) and a hydrophilic protective polymer (PEG3K). The PRN can recognize and selectively bind to overexpressed Hpn, which results in enhanced internalization via receptor-mediated endocytosis. The PRN escapes from the endosomes and releases DTX and TRQ into the cytosol. Subsequently, TRQ inhibits the P-glycoprotein efflux pump and thereby enhances the intracellular accumulation of DTX to induce cancer cell apoptosis. Compared to the dual-carrier system, the single-carrier system could, due to increased delivery efficiency by co-encapsulation, induce a stronger multidrug-resistance-reversal effect.

In this study, we evaluated whether the simultaneous delivery of DTX and TRQ using a PRN (single-type nanocarrier) would increase the therapeutic efficacy of DTX by reversing the MDR effect in drug-resistant breast cancer cells as compared with delivery using dual-type nanocarriers. As illustrated in Figure 1, using the well-established PRN system, both DTX and TRQ were delivered to the drug-resistant cancer MCF7/ADR cells. We hypothesized that the single-type nanocarrier system could exhibit stronger P-gp inhibition than the dual-carrier system owing to the increased delivery efficiency conferred by co-encapsulation. We determined the nanoparticulate characteristics, including the co-loading capacity, physical stability on storage, and drug-release profile. The potential of MDR reversal was evaluated by analyzing the in vitro antitumor effects in terms of cytotoxicity, apoptotic cell death, and the cell-cycle distribution of the cells. The internalization efficiency between the dual-type and single-type nanocarriers was compared via the competitive cellular uptake assay. Finally, in vivo antitumor efficacy was assessed using mice bearing MCF7/ADR tumor xenografts.

## 2. Results and Discussion

### 2.1. Determination of Drug Encapsulation Capacity of DTX- and TRQ-Loaded PRN (D^T-PRN)

**Figure 2:**
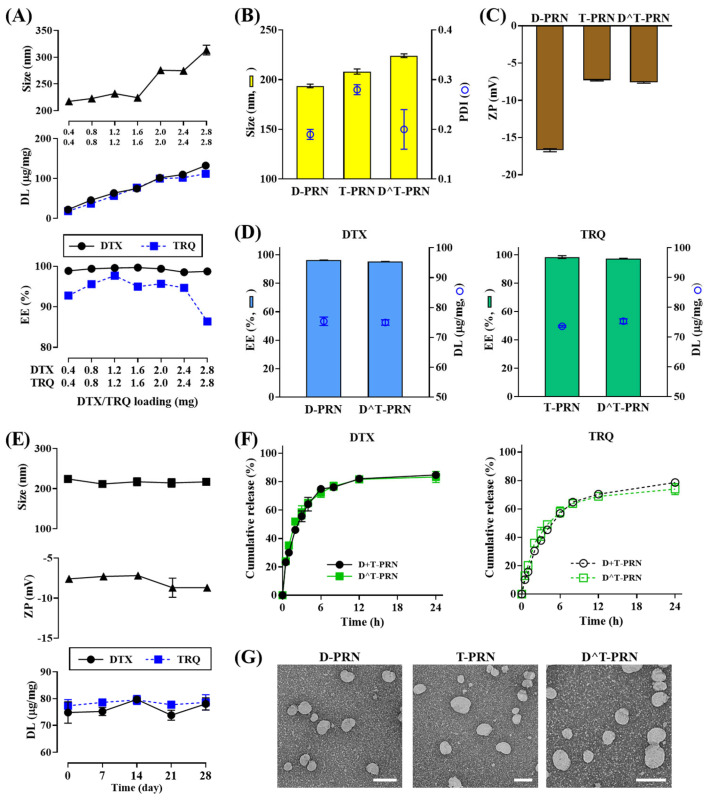
Characteristics of docetaxel (DTX)- and/or tariquidar (TRQ)-loaded PEGylated (5 mol%) and RIPL peptide-conjugated nanostructured lipid carrier (PRN) samples. (**A**) Determination of the particle size, drug-loading (DL) capacity, and encapsulation efficiency (EE) of DTX- and TRQ-loaded PRN (D^T-PRN). The ratio of DTX and TRQ was fixed as 1:1 (*w*/*w*), and the amounts of DTX and TRQ ranged from 0.4 to 2.8 mg. (**B**) Particle sizes of the drug-loaded PRN samples. (**C**) Zeta potential (ZP) of drug-loaded PRN samples. (**D**) EE and DL capacity of drug-loaded PRN samples. (**E**) Physical stability of D^T-PRN stored at 4 °C for 4 weeks. (**F**) In vitro release profiles of DTX and TRQ from the physically mixed dual-carrier system of DTX-loaded PRN (D-PRN) and TRQ-loaded PRN (T-PRN) (D+T-PRN) or D^T-PRN. (**G**) Transmission electron microscopy images. Scale bar = 200 nm. Data represent the mean ± standard deviation (SD) (*n* = 3).

The maximum amount of DTX and TRQ that can be loaded was determined by increasing the content of loaded drugs, while maintaining the ratio of DTX and TRQ at 1:1 (*w*/*w*) in the preparation of D^T-PRN. The ratio of DTX and TRQ at 1:1 (*w*/*w*), which showed the strongest synergism with the lowest combination index (CI) value, based on the data from a previous study [25], was applied as the optimal ratio. The added DTX and TRQ content with the fixed ratios varied from 0.4 to 2.8 mg. As shown in Figure 2A, the increase in the DTX and TRQ drug content in D^T-PRN did not influence the particle size of D^T-PRN until a 1.6 mg concentration of each drug was attained. However, when the amount of DTX and TRQ in D^T-PRN increased to 2 mg each, the particle size of D^T-PRN increased to approximately 300 nm. Both DTX and TRQ showed more than 95% encapsulation efficiency (EE), and the ratio between DTX and TRQ (1:1, *w*/*w*) was maintained until a 2.0 mg concentration of each drug was reached. An increase in the nanocarrier size may affect the extravasation and accumulation within the tumor tissue through multiple mechanisms, including the enhanced permeability and retention (EPR) effect, internalization into cells, or nanocarrier stability [26,29]. Thus, a dose of 1.6 mg was selected as the amount of each drug for optimal co-loading of D^T-PRN for subsequent experiments and was used to prepare DTX-loaded PRN (D-PRN) or TRQ-loaded PRN (T-PRN).

### 2.2. Characterization of Different PRNs 

The physicochemical characteristics of D-PRN, T-PRN, and D^T-PRN were evaluated in terms of particle size, polydispersity index (PDI), zeta potential (ZP), EE, drug-loading (DL), drug release, and morphology. The average sizes of the PRN formulations ranged from 193 to 224 nm, as measured by dynamic light scattering (DLS) (Figure 2B). There were no significant changes in the particle size after single-drug encapsulation of DTX or TRQ, whereas the particle size of D^T-PRN slightly increased. All formulations had PDI values of less than 0.3, indicating a homogenous and narrow particle size distribution. ZP measurements revealed that the PRN samples were negatively charged; however, the absolute values of ZP showed a change, depending on the type of encapsulated drug (Figure 2C). Drug-free PRN had a ZP value of –15 to –17 mV (data not shown), similar to that of D-PRN. The ZP value was −16.7, −7.3, and −7.6 mV for D-PRN, T-PRN, and D^T-PRN, respectively. We assumed that TRQ molecules were not only partitioned throughout the matrix, but also distributed in the vicinity of the surface. The neutralized ZP values by TRQ loading may be attributable to the positively charged amine groups in the drug moiety [30,31]. The EE and DL values were 95.7%–98.4% and 73.6–75.4 μg/mg, respectively, for DTX- and/or TRQ-loaded PRN formulations (Figure 2D) and did not significantly differ between PRNs prepared by the simultaneous loading of DTX and TRQ. 

The physical stability of D^T-PRN was verified over 4 weeks at 4 ± 1 °C by analyzing the changes in particle size, PDI, ZP, and DL. As shown in Figure 2E, D^T-PRN remained stable, without any significant change in physicochemical properties, including particle size, ZP, and DL over the period of storage. On Day 28, the particle size of D^T-PRN did not significantly differ and remained approximately 220 nm (PDI < 0.3; data not shown). The ZP of D^T-PRN also remained unchanged as compared to the initial values, and the DL of D^T-PRN was also maintained. Further, in our earlier reports, we found that the PRN system was stable in the serum containing media, without significant changes in physicochemical properties at 37 °C for 24 h [24,25]. Consequently, the co-loading of DTX and TRQ did not affect the stability of D^T-PRN, and the colloidal stability remained unaltered over the 4-week storage period, without signs of particle aggregation and drug leakage until its use. 

The cumulative release profiles of DTX or TRQ from the single- and dual-carrier systems are shown in Figure 2F. Regardless of the carrier type, the drug release was biphasic, showing no difference between the systems. DTX release from the dual and single systems was approximately 64.2% and 65.3% at 4 h, respectively, and plateaued after 12 h. In contrast, the release of TRQ from the dual and single systems was 45.3% and 49.0% at 6 h, respectively, which slowly increased to ~74% at 24 h. Drug-loaded PRN showed a prolonged and sustained drug-release pattern after the initial rapid release, which was possibly attributable to the step-wise drug release from NLC. There was an initial fast release of the drug adsorbed and/or adjacently located on the surface, followed by sustained drug release from the internal lipid core due to diffusion and erosion of the matrix [32,33]. As shown in Figure 2G, transmission electron microscopy (TEM) images showed that all prepared PRNs comprised well-dispersed nano-sized particles (<230 nm) with a homogeneous spherical morphology, and this indicated their suitability for systemic administration [34,35]. Thus, we expect that D^T-PRN can accumulate in tumor tissues by the EPR effect and thus become readily internalized into cancer cells via receptor-mediated endocytosis [36]. Moreover, their sustained release pattern would reduce the frequency of drug administration and be beneficial for clinical anticancer therapy.

### 2.3. Cytotoxicity

**Figure 3:**
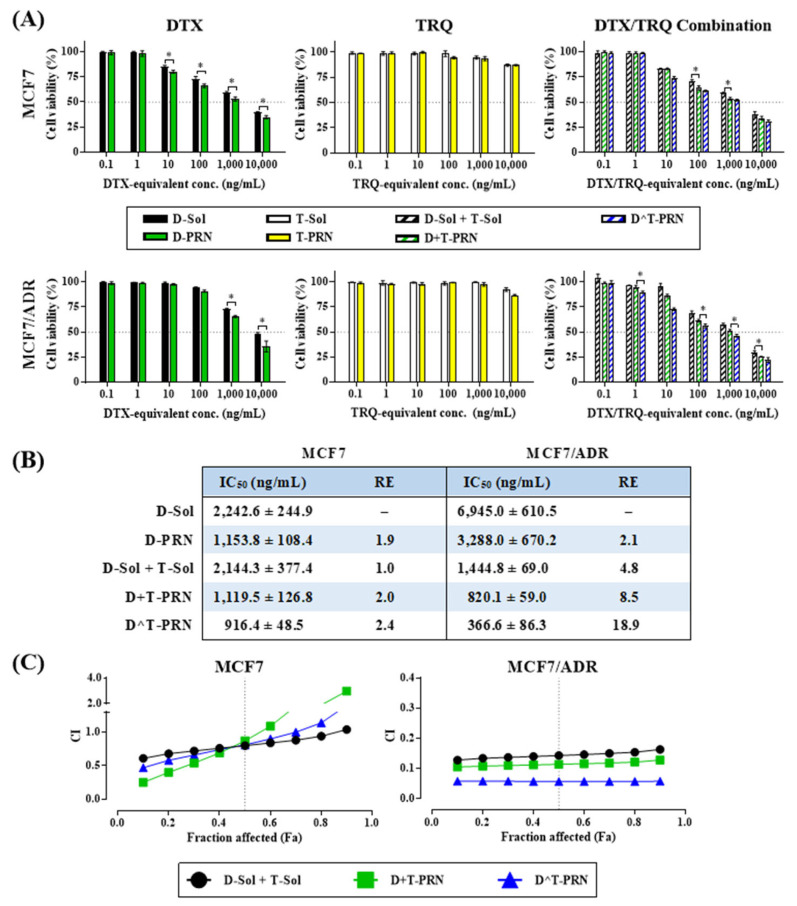
In vitro cytotoxicity studies of different formulations in MCF7 and MCF7/ADR cells. (**A**) Cell viability was evaluated by the water-soluble tetrazolium salt-1 (WST-1) assay. Statistical analysis was performed using a Student’s t-test (* *p* < 0.05). (**B**) Half-maximal inhibitory concentration (IC50; docetaxel [DTX]-equivalent) and reversal efficiency (RE) values of different DTX-loaded formulations. (**C**) A plot of the combination index (CI) against the fraction affected (Fa). Data represent the mean ± standard deviation (SD) (*n* = 3).

The in vitro cytotoxicity of the various formulations that were prepared was tested in MCF7 (drug-sensitive) and MCF7/ADR (drug-resistant) cells using the water-soluble tetrazolium salt-1 (WST-1) assay (Figure 3A). All DTX-loaded formulations or co-treatments exhibited concentration-dependent cytotoxicity in both cell lines. There was a gradual increase in cytotoxicity at concentrations above the DTX-equivalent concentration of 10 ng/mL for MCF7 cells or 100 ng/mL for MCF7/ADR cells. In contrast, the TRQ-only formulations were not cytotoxic up to a TRQ-equivalent concentration of 1000 ng/mL in both cell lines. However, a slight decrease of 5–10% in cell viability was observed at concentrations above 10,000 ng/mL. Combined treatment with TRQ and DTX did not significantly increase the cytotoxicity of DTX in drug-sensitive MCF7 cells. However, the combination increased the cytotoxic effect of DTX in MCF7/ADR cells, and the cell viability was lowered at DTX-equivalent concentrations above 10 ng/mL.

The values of half-maximal inhibitory concentration (IC_50_), reversal efficiency (RE), and CI at a fraction affected (Fa) of 0.5 for the different DTX-loaded formulations and the DTX–TRQ combination treatments are shown in Figure 3B. The extent to which MDR is overcome was determined based on RE, which is the ratio of IC_50_ of D-Sol to that of the drug-loaded formulations. The IC_50_ values of the DTX-loaded formulations were observed in the following order: D^T-PRN < the physically mixed dual-carrier system of D-PRN and T-PRN (D+T-PRN) ≤ D-PRN < the mixture of DTX-dissolved solution (D-Sol) and TRQ-dissolved solution (T-Sol) (DTX:TRQ = 1:1, *w*/*w*) (D-Sol+T-Sol) ≤ D-Sol in MCF7 cells; D^T-PRN << D+T-PRN < D-PRN < D-Sol + T-Sol << D-PRN << D-Sol in MCF7/ADR cells. To achieve the same level of cytotoxic effect, MCF7/ADR cells required a higher dose of DTX than MCF7 cells due to P-gp overexpression [37]. In both cell lines, D-PRN showed a twofold lower IC_50_ value than D-Sol. The superiority of PRN formulations over the Sol system is attributed to the active targeting capability of PRN that is modulated via RIPL peptide-mediated transport, which subsequently increases the intracellular drug concentration [25,38].

When the IC_50_ values observed in the absence and presence of TRQ treatment were compared, the cytotoxic effect of the DTX and TRQ combination differed in the MCF7 and MCF7/ADR cells due to the different expressions of P-gp. In MCF7 cells, D-Sol + T-Sol or D+T-PRN did not significantly reduce the IC_50_ value compared to that observed with D-Sol or D-PRN (single DTX treatments). Furthermore, D^T-PRN showed the lowest IC_50_ value. For comparison, when converted to RE, the PRN systems (D-PRN, D+T-PRN, and D^T-PRN) showed slightly increased values of 1.9, 2.0, and 2.4, respectively. In contrast, in MCF7/ADR cells, DTX with TRQ showed greatly increased cytotoxicity. The IC_50_ values of D-Sol + T-Sol and D+T-PRN were approximately four times lower than those of D-Sol and D-PRN, respectively. In particular, D^T-PRN showed the lowest IC_50_ value, which was 2.2-fold lower than that of D+T-PRN. As a result, D^T-PRN revealed an RE value of 18.9, which was 9.0, 3.9, and 2.2 times higher (statistically significant) than those of D-PRN, D-Sol + T-Sol, and D+T-PRN, respectively. 

Accordingly, we interpreted that the excellent cytotoxicity of the combination in MDR cells was due to the P-gp inhibition. At Fa = 0.5, D+T-PRN and D^T-PRN showed CI values close to 0.8 (moderate slight synergism) in MCF7 cells, whereas the values in MCF7/ADR cells were approximately 0.11 (strong synergism) and 0.05 (very strong synergism), respectively (Figure 3C). The greatest cytotoxic effect of D^T-PRN in MDR cancer cells is attributed to the effect of targeted nanoparticles that can enhance DTX accumulation [39]. Some mechanisms were proposed with regard to the MDR reversal activity of nanoparticles via the bypassing of P-gp-mediated efflux [28]. Moreover, D^T-PRN is a co-encapsulated single-carrier system that might exhibit consistent cellular uptake behavior compared to the dual-carrier system during the internalization process. Thus, D^T-PRN was endocytosed into cells in a receptor-mediated manner without any interference and simultaneously released its cargo [40].

### 2.4. Cell-Cycle Analysis

**Figure 4:**
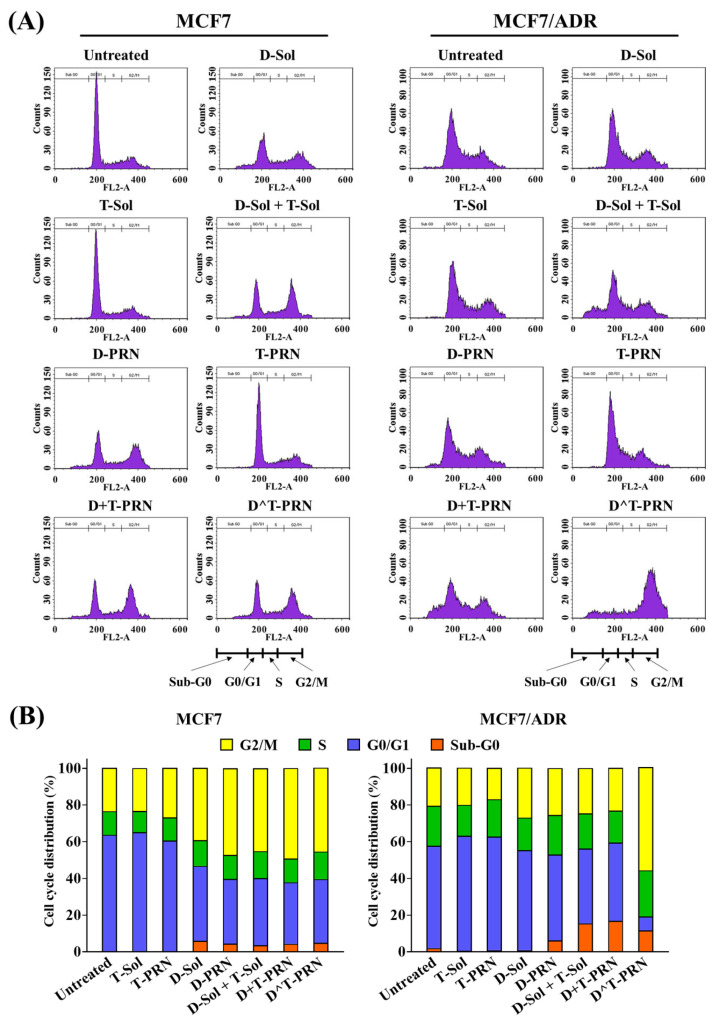
Effect of 24 h treatment with docetaxel (DTX)- and/or tariquidar (TRQ)-loaded formulations on the cell cycle of MCF7 and MCF7/ADR cells. (**A**) FACS histogram of the cell cycle and (**B**) quantitative analysis of cell-cycle arrest.

DTX induces G2/M phase arrest in mitosis and subsequent apoptosis [1,3]. Figure 4 illustrates the change in the cell-cycle distribution after treatment with various drug-loaded samples. The effects of free drugs and drug-loaded PRN samples on the distribution in the cell cycle for MCF7 and MCF7/ADR cells were evaluated by analyzing the propidium iodide (PI)-stained cellular DNA content by flow cytometry. Most of the untreated cells exhibited the cell-cycle distribution in the G0/G1 phase, and approximately 23.54% and 20.82% of the MCF7 and MCF7/ADR cells were captured in the G2/M phase, respectively. As expected, both cell lines treated with TRQ-only formulations (T-Sol and T-PRN) were mainly accumulated in the G0/G1 phase, but without any increased cell population in the phase of the Sub-G0 or G2/M. In contrast, all DTX treatments significantly decreased the cell populations of the G0/G1 phase and increased those of the G2/M or Sub-G0 phases.

As shown in the bar graphs (Figure 4B), the MCF7 cell population in the G2/M phase increased to 36.4% and 47.3% after incubation with D-Sol and D-PRN, respectively. A similar distribution pattern in the cell cycle was observed in MCF7 cells after the co-treatment with DTX and TRQ, regardless of the treatment group. Compared with untreated cells, DTX-only formulation-based treatment of MCF7/ADR cells induced a slight increase in the cell population in the G2/M phase. The percentage of MCF7/ADR cells arrested in the G2/M phase after treatment with D-Sol and D-PRN increased to 27.2% and 25.6%, respectively. Importantly, compared to DTX-only treatment, combination therapy with D-Sol + T-Sol and D+T-PRN induced G2/M phase arrest (24.7% and 23.3, respectively) and resulted in significantly higher cell populations in the Sub-G0 phase (15.4% and 16.9%, respectively). The treatment of D^T-PRN markedly increased the accumulation of cell populations in the G2/M phase to 56.2%. DTX induces microtubule stabilization that leads to the cell-cycle arrest in the G2/M phase and subsequent apoptotic cell death [41]. In MCF7 cells, TRQ did not show any direct cytotoxicity. However, in the MCF7/ADR cells, the cell population in the G2/M phase increased in the D^T-PRN-treated groups, which was due to the P-gp-inhibiting effect of TRQ that increased the cytoplasmic DTX concentration [38].

### 2.5. Cell Apoptosis Analysis

**Figure 5:**
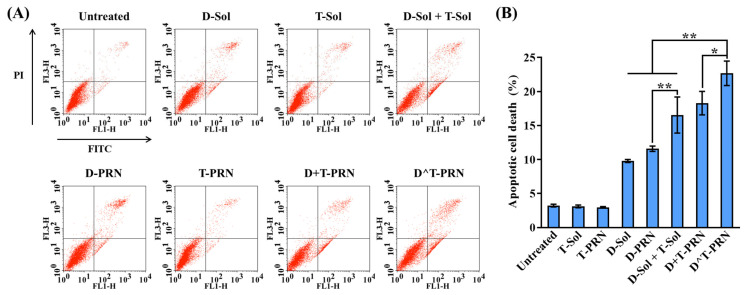
Induction of apoptosis in MCF7/ADR cells following 24 h treatment with docetaxel (DTX)- and/or tariquidar (TRQ)-loaded formulations at a 10 ng/mL DTX-equivalent concentration. (**A**) Scatter plots and (**B**) quantitative cells; top right (Q2, FITC+/PI+), late apoptotic cells; bottom left (Q3, FITC–/PI–), live cells; and bottom right (Q4, FITC+/PI–), early apoptotic cells. Data represent mean ± standard deviation (SD) (*n* = 3). The differences were significant at * *p* < 0.005 and ** *p* < 0.001.

Enhanced cell apoptosis in MCF/ADR cells was evaluated quantitatively using the Annexin V-fluorescein isothiocyanate (FITC)/PI double-staining assay. The flow cytometric quadrantal diagram in Figure 5A is divided into four parts to facilitate the interpretation of the apoptotic tendency. The bottom left (Q1; Annexin–/PI–), bottom right (Q2; Annexin+/PI–), top left (Q3; Annexin–/PI+), and top right (Q4; Annexin+/PI+) quadrants indicate viable cells, early apoptotic cells, late apoptotic cells, and necrotic cells, respectively. Figure 5B represents the percentage of early and late apoptotic MCF7/ADR cell populations among the experimental cell populations. A very low apoptosis level was detected in the untreated controls (3.23 ± 0.21%) and TRQ-only formulations (<4%). A one-way independent-groups analysis of variance (ANOVA) was performed to assess whether different treatments influenced the extent of cell death and yielded a significant effect of treatment on cell apoptosis (F(6,14) = 125.89, *p* < 0.001), which suggested that the extent of apoptosis differed by treatment type (Supplementary Material Table S1).

A post hoc Tukey’s test showed that both T-Sol (mean (M) = 3.11) and T-PRN (M = 2.98) did not differ significantly from the untreated group (M = 3.23). In comparison, the DTX-containing formulations significantly increased the total apoptosis rates. The apoptosis rate of cells treated with D-PRN (M = 11.59) differed significantly from that of cells in the other groups, except D-Sol (M = 9.80). This indicated that DTX was the only relevant treatment that significantly increased cell death. Meanwhile, D^T-PRN (M = 22.67) and D+T-PRN (M = 18.30) were significantly more effective than all of the remaining treatment groups, including the untreated or control group. This suggested that either the single or dual system considerably improved the rate of apoptosis, compared to any single-drug treatment type. Moreover, D^T-PRN was the most prominent drug delivery system, with a significantly greater extent of apoptosis than that achieved with D+T-PRN; that is, the single-delivery system (D^T-PRN) was preferable for apoptosis over the dual-delivery system. This outcome might be attributed to the different cellular uptake behaviors between the dual- and single-carrier systems, which result in an improper drug concentration ratio in cancer cells [25,26].

### 2.6. Competitive Cellular Uptake

The surface modification of nanocarriers with targeting ligands that can recognize the overexpressed receptors on cancer cells and enhance the internalization via ligand–receptor binding has been an effective strategy for tumor targeting [42,43]. We previously developed a Hpn-specific PRN system and demonstrated the enhanced internalization of PRN via receptor-mediated endocytosis (RME) [21]. Moreover, we investigated the potential of the competitive cellular uptake patterns of dual and single systems using PRN for multidrug delivery.

**Figure 6:**
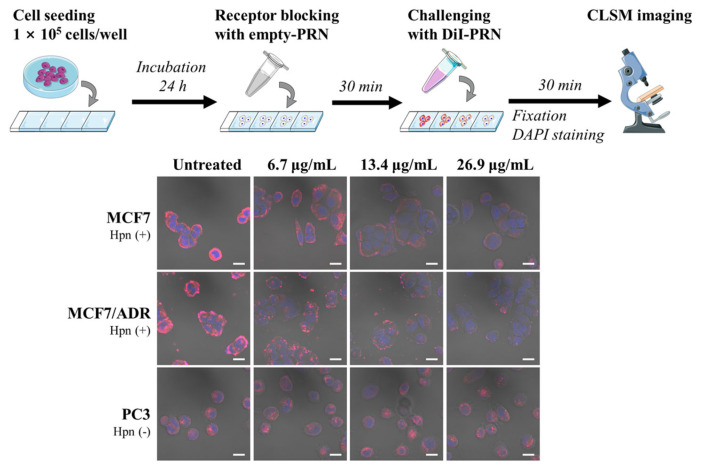
Cellular uptake of 1,1′-dioctadecyl-3,3,3′,3′-tetramethylindocarbocyanine perchlorate (DiI)-loaded PEGylated (5 mol%) and RIPL peptide-conjugated nanostructured lipid carrier (PRN; DiI-PRN) after hepsin (Hpn) pre-blocking in Hpn-positive cells (MCF7 and MCF7/ADR) and Hpn-negative cells (PC3) by CLSM. Cells were pretreated with empty-PRN (lipid concentration of 0, 6.7, 13.4, and 26.9 μg/mL) for 30 min to ensure receptor blocking, followed by treatment with DiI-PRN (DiI-equivalent concentration of 25 ng/mL) at 37 °C for 30 min. The nucleus was stained with 4′,6-diamidino-2-phenylindole (DAPI) for blue fluorescence and merged with the red fluorescence of cytoplasmic DiI. Scale bar = 10 μm.

As illustrated in Figure 6, changes in the cellular uptake efficiency were observed, according to the degree of receptor blockade. Hpn was blocked by pretreatment with empty-PRN for 30 min, and the cells were treated with an equivalent amount of 1,1′-dioctadecyl-3,3,3′,3′-tetramethylindocarbocyanine perchlorate (DiI)-loaded PRN (DiI-PRN). Confocal laser scanning microscopy (CLSM) showed drastically decreased cellular uptake of DiI-PRN in Hpn-positive cells (MCF7 and MCF7/ADR) as the amount of treated empty-PRN increased. However, the uptake by Hpn-negative PC3 cells was not dependent on the empty-PRN pretreatment. As previously reported, PRN is internalized by RME after the interaction between the IPL sequence of the RIPL peptide on the surface of PRN and Hpn [19,20]. When the receptors and ligands are bound, the number of free target receptors is limited. Subsequently, the nanoparticles compete to occupy the unbound target receptors [44,45]. This competition interferes with the transport of the nanoparticles into the cytoplasm and subsequently reduces the delivery efficiency of ligand-modified nanoparticles. 

**Figure 7:**
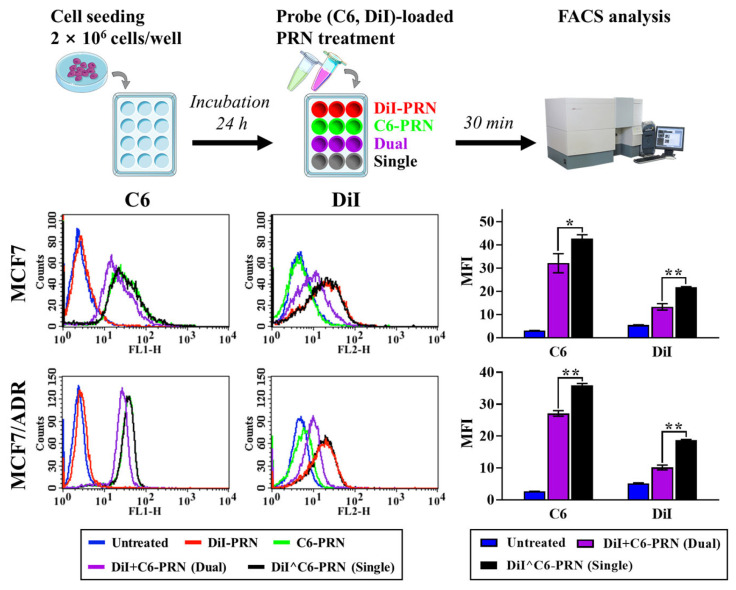
FACS analysis of the competitive cellular uptake for the comparison of the probe-loaded dual-carrier system with the single-carrier system. The cells were incubated for 30 min at 37 °C in a medium containing a physically mixed dual-carrier system of 1,1′-dioctadecyl-3,3,3′,3′-tetramethylindocarbocyanine perchlorate (DiI)-loaded PEGylated (5 mol%) and RIPL peptide-conjugated nanostructured lipid carrier (PRN; DiI-PRN) and coumarin-6 (C6)-loaded PRN (C6-PRN) (DiI+C6-PRN; Dual delivery system) or DiI- and C6-loaded PRN (DiI^C6-PRN; Single delivery system) at the following concentrations: DiI: 50 ng/mL; C6: 16.4 ng/mL. Data represent the mean ± standard deviation (SD) (*n* = 3). Significant differences were noted at * *p* < 0.005 and ** *p* < 0.001.

The competitive cellular uptake behavior after different PRN treatments was evaluated using two types of fluorescence probes, coumarin-6 (C6) and DiI, exhibiting green and red fluorescence and replacing TRQ and DTX, respectively. C6-loaded PRN (C6-PRN) and DiI-PRN were used as positive controls. In both Hpn-positive MCF7 and MCF7/ADR cells, the DiI- and C6-loaded PRN (DiI^C6-PRN; single) delivered both fluorescent probes simultaneously and uniformly to the same target population of cells (Figure 7). A one-way independent-groups ANOVA followed by post hoc Tukey’s test was performed to examine the significant differences in cellular uptake behavior between the dual- and single-carrier systems. ANOVA yielded a significant effect of the single system on cellular uptake, without competition between nanocarriers: (F(2,6) = 218.62, *p* < 0.001) for C6 in MCF7, (F(2,6) = 235.11, *p* < 0.001) for DiI in MCF7, (F(2,6) = 1011.89, *p* < 0.001) for C6 in MCF7/ADR, and (F(2,6) = 821.96, *p* < 0.001) for DiI in MCF7/ADR (Supplementary Material Table S2). Post hoc Tukey’s test showed that the single-carrier system significantly increased the fluorescence intensity of both probes in both cell lines compared to the dual-carrier system (*p* < 0.001). The reduced cellular uptake behavior of the dual-carrier system is potentially attributable to the possibility that the same nanocarriers can interfere with each other’s uptake by competing for receptor binding [26,38]. Gao et al. showed through real-time imaging that this self-competition with the dual-nanocarrier system appeared immediately after the cells were treated with the nanoparticles [40]. Even if the receptor-binding competition continues, the single-carrier system that co-encapsulates multiple drugs can deliver its cargo simultaneously to the target cell while maintaining the optimal ratio [46]. Therefore, we concluded that the single system is more suitable for delivering a combination of drugs to exert their effects at the same time and in the same space, such as DTX and TRQ.

### 2.7. In Vivo Antitumor Efficacy

**Figure 8:**
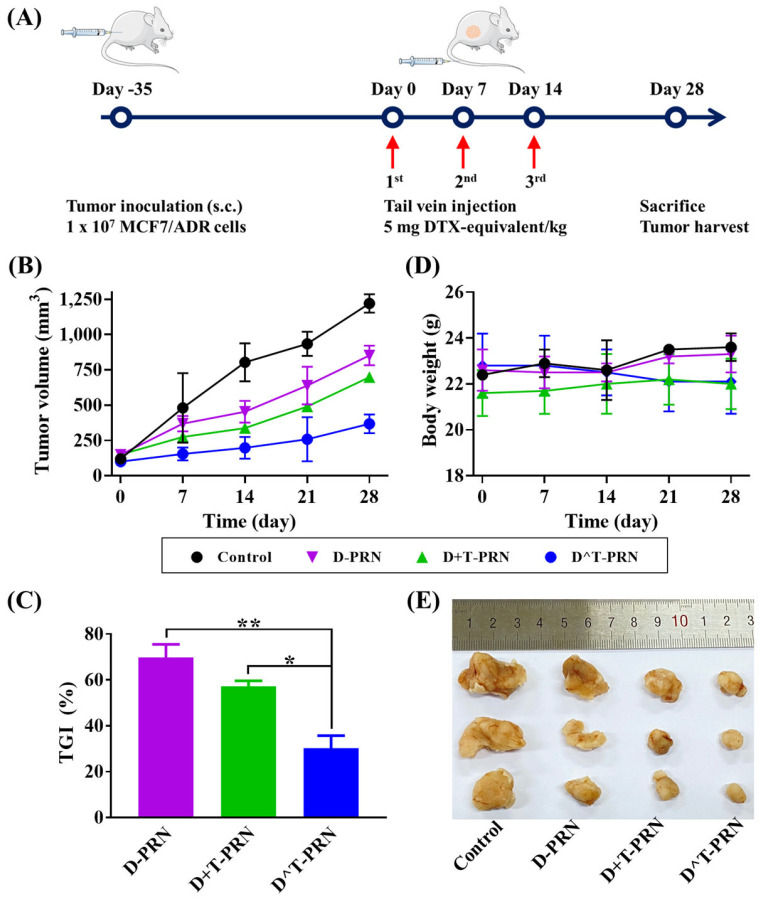
In vivo antitumor efficacy in an MCF7/ADR-xenograft mouse model. (**A**) Schematic representation of the in vivo treatment schedule after the tail vein injection of different formulations at a 5 mg docetaxel (DTX)-equivalent/kg dose on days 0, 7, and 14, for a total of three injections per mouse. (**B**) Changes in tumor volume over 28 days after administration. (**C**) Tumor growth inhibition (TGI) on Day 28. (**D**) Changes in body weight. (**E**) Morphology of excised xenograft tumors at the end of the study (Day 28). Data are the mean ± SD (*n* = 3). The differences were significant at * *p* < 0.005 and ** *p* < 0.001.

A BALB/c MCF7/ADR-bearing xenograft mouse model was established 5 weeks after subcutaneous inoculation of the MCF7/ADR cell lines to determine the in vivo antitumor activity of the various tested formulations. MCF7/ADR-bearing xenograft mice were randomly divided into four groups: Group 1—control (saline only), Group 2—D-PRN, Group 3—D+T-PRN, and Group 4—D^T-PRN. Mice were treated as depicted in Figure 8A. The tumor grew exponentially in all groups (Figure 8B). Compared to the control group, drug-laden PRN formulations inhibited tumor growth after the second administration. D-PRN exhibited relatively greater tumor growth inhibition than the control. In comparison to the weak tumor-suppressive effect of D-PRN, D+T-PRN showed considerable tumor growth inhibition after Day 7. The D^T-PRN treatment showed higher antitumor efficacy than that of the other treatment groups. At the end of the experiment (Day 28), tumor volumes reached approximately 1220, 851, 698, and 368 mm^3^ in the control, D-PRN, D+T-PRN, and D^T-PRN treatment groups, respectively. In particular, the D^T-PRN-treated group exhibited the strongest inhibitory effect on MCF7/ADR tumor growth, showing 2.3- and 1.9-times greater inhibition compared with the D-PRN and the D+T-PRN groups, respectively. 

The suppression of tumor volumes is expressed as a tumor growth inhibition (TGI) percentage (Figure 8C). A one-way independent-groups ANOVA followed by the post hoc Tukey’s test was performed to examine the significant differences among the treatment groups. ANOVA yielded a significant effect of treatments on TGI (%) (F(2, 6) = 53.64, *p* < 0.001; Supplementary Material Table S3). Post hoc Tukey’s test showed that D^T-PRN (M = 30.16%) was significantly more effective than D-PRN (M = 69.77%) and D+T-PRN (M = 57.18%).

As depicted in Figure 8D, the body-weight changes of tumor-bearing mice were monitored as an indication of safety and revealed no significant intergroup differences in body weight in the treatment groups. As the tumor grew substantially, the mice of the control group exhibited behavioral changes, including impaired movement and skin dryness, whereas no obvious change was observed in the D-PRN treatment and DTX and TRQ combination treatment groups. At the end of the experiment, the mice were sacrificed, and the tumors were excised and photographed for further comparison (Figure 8E). The image of the tumor tissues of the test group further confirmed the efficient tumor growth inhibition by D^T-PRN.

Combining P-gp inhibitors to overcome drug resistance that is mediated by efflux transporters has been widely used to increase the therapeutic efficacy of DTX by restoring the sensitivity of the tumor cells [8,47]. Pan et al. synthesized cancer stem cells (CSCs)-specific, targeted, mesoporous, silica-based nanocarriers for co-delivery of doxorubicin and tariquidar to eliminate the CSCs and overcome the MDR of breast CSCs [48]. Diao et al. constructed a CD44-targeted, self-assembled NP system that contains curcumin and doxorubicin to treat drug-resistant tumor cells through combination therapy [49]. The simultaneous delivery of two or more drugs to the target cells, using a nanoparticulate system, is expected to be a more efficient approach than the drug cocktail therapy used currently [50].

The D^T-PRN treatment group showed greater antitumor efficacy than the other treatment groups due to the following advantages. First, NLCs have a high payload capacity for multiple drugs and can achieve the subsequent sustained release of the cargos [51]. Encapsulation of DTX and TRQ in NLCs can protect them from the metabolic degradation and immature clearance from circulation before reaching the target site [52] and can improve the pharmacokinetics and biodistribution profiles of the loaded drugs by virtue of the high surface-to-volume ratio of NLCs [50,51]. In addition, NLCs may be extravasated and thereby accumulate in the surrounding tumor tissue owing to their ideal nano-size of approximately 200 nm [52,53].

Second, the superiority of the D^T-PRN could be attributed to the improved passive and active targeting capability of PRN via surface functionalization. The designing of PRN using 1 mol% of Hpn-specific RIPL peptide and 5 mol% PEG3K conferred in vivo stability and biodistribution to the nanocarrier [21,23]. The presence of the hydrophilic protective polymer–PEG on the surface of PRN may reduce the capture by the mononuclear phagocyte system, enhance in vivo stability, and prolong the circulation time of PRN, leading to increased tumor accumulation by passive targeting [54,55,56]. Moreover, with the RIPL peptide, PRN could selectively bind to Hpn-expressing cancer cells and enhance the intracellular co-delivery of DTX and TRQ following their distribution and penetration of tumor tissues [20,35]. In particular, compared to the dual system that undergoes competitive receptor-ligand binding, the single system can achieve the spatiotemporal delivery of two drugs by simultaneous loading [40].

Third, for an efficient combination chemotherapy, DTX and TRQ should be simultaneously delivered intracellularly, while maintaining the optimized drug ratio that has demonstrated strong synergism [57]. As explained above, D^T-PRN specifically binds to Hpn-expressing cells, including MDR cancer cells, and is then internalized into the cell by RME. After achieving endosomal escape, D^T-PRN can bypass P-gp efflux pumps and release the optimal fixed ratio of DTX and TRQ into the cytosol [28]. Subsequently, TRQ inhibits the P-gp efflux pump and enhances DTX accumulation in the drug-resistant cells [11,58]. The retained DTX triggers an apoptotic cascade by inducing of G2/M phase arrest and can thereby enhance cell death [59,60,61]. Altogether, our results indicate the superior anticancer efficacy of the combination of DTX and TRQ using D^T-PRN against chemoresistant breast cancer.

## 3. Materials and Methods

### 3.1. Materials

DTX (>99% purity) was kindly provided by Chong Kun Dang Pharm. Co. (Yongin, Republic of Korea). TRQ (>97.5% purity) was purchased from MedChemExpress (Monmouth Junction, NJ, USA). Oleoyl macrogol-6 glycerides (Labrafil^®^ M 1944 CS) and glyceryl distearate (Precirol^®^ ATO 5) were gifted by Gattefossé (Saint-Priest, France). 1,2-Distearoyl-sn-glycero-3-phosphoethanolamine-*N*-[methoxy(polyethylene glycol_3000_)] (DSPE-PEG3K) and DSPE-*N*-[maleimide(polyethylene glycol_2000_)] (DSPE-PEG2K-Mal) were purchased from Avanti Polar Lipids (Alabaster, AL, USA). DiI, C6, phosphate-buffered saline (PBS) tablets, and Accutase^®^ solution were purchased from Sigma-Aldrich Chemical Co. (St. Louis, MO, USA). EZ-cytox WST-1 reagent solution was purchased from Dail Lab service (Seoul, Republic of Korea). The RIPL peptide was synthesized by Peptron Co. (Daejeon, Republic of Korea). Cell culture materials, including PBS (10×, pH 7.4), Roswell Park Memorial Institute (RPMI) 1640 medium, fetal bovine serum, and penicillin–streptomycin, were obtained from Invitrogen (Carlsbad, CA, USA). BD Matrigel^TM^ basement membrane matrix was obtained from BD Biosciences (San Jose, CA, USA). All other chemicals and reagents that were purchased from commercial sources were of analytical or cell culture grade.

### 3.2. Cell Culture and Animals

The human breast adenocarcinoma (MCF7) and human prostate adenocarcinoma (PC3) cell lines were purchased from the Korean Cell Line Bank (Seoul, Republic of Korea). Drug-resistant MCF7 cells (MCF7/ADR) were kindly provided by Prof. Dr. Kyung Hoon Min (College of Pharmacy, Chung-Ang University, Seoul, Republic of Korea). Cell lines were grown in RPMI 1640 medium supplemented with 100 μg/mL streptomycin, 100 U/mL penicillin G, and 10% (*v*/*v*) fetal bovine serum. Cells were subcultured every 3 to 5 days in a humidified atmosphere of 5% CO_2_ at 37 °C and 95% relative humidity. BALB/c athymic mice (17 ± 2 g, female, 6 weeks old) were purchased from the Hanlim Experimental Animal Laboratory (Gyeonggi-do, Republic of Korea).

### 3.3. Synthesis of DSPE-PEG2K-RIPL

The RIPL peptide was conjugated with DSPEG-PEG2K-Mal by a thiol-maleimide reaction between the maleimide group of DSPE-PEG2K-Mal and the sulfhydryl side chain of the RIPL peptide’s cysteine residue, according to a previously reported method [62]. Briefly, DSPE-PEG2K-Mal and RIPL peptides were dissolved in 0.01 M PBS (pH 7.4) at a molar ratio of 1:1.12. After stirring in the dark for 48 h at 25 °C, the reaction mixture was dialyzed against double-distilled water to remove the unconjugated RIPL peptides, using a 3.5 kDa MWCO dialysis membrane (Biotech CE Tubing; Spectrum Laboratories, Inc., Rancho Dominguez, CA, USA) at 4 °C for 24 h. The final purified solution was then recovered, lyophilized at −45 °C under 8 Pa pressure overnight using a freeze-dryer (FDU-1200; EYELA, Miyagi, Japan), and stored at 4 °C until use.

### 3.4. Preparation of Reference Solutions

To mimic a commercial DTX formulation (Taxotere^®^), D-Sol was prepared and used as the reference sample [63]. Briefly, DTX was formulated in sterilized double-distilled water containing 25% (*w*/*v*) Tween 80 and 9.75% (*v*/*v*) ethanol to obtain a concentration of 10 mg/mL. To prepare the T-Sol, TRQ was dissolved in dimethyl sulfoxide (DMSO) at 5 mg/mL. For in vitro and in vivo experiments, D-Sol and T-Sol were appropriately diluted with the cell culture medium or normal saline. The final concentration of DMSO in the culture was less than 0.2%, which is nontoxic to cells.

### 3.5. Preparation of PRN Formulations

The PRN formulations were constituted by a solvent emulsification–evaporation method, as reported previously [22], and were divided into D-PRN, T-PRN, and D^T-PRN groups. To prepare the D^T-PRN, the ratio of DTX and TRQ was fixed as 1:1 (*w*/*w*), and the added amounts of DTX and TRQ were varied (0.4–2.8 mg, respectively) to determine the DL capacity. Briefly, to prepare D^T-PRN, an aqueous phase containing Tween 20 (1%, *w*/*v*), polyvinyl alcohol (0.5%, *w*/*v*), and 1 mol% DSPE-PEG2K-RIPL was incorporated into the organic phase prepared by dissolving DTX, TRQ, Labrafil^®^ M 1944 CS (6.2 μL), Precirol^®^ ATO 5 (15.3 mg), and 5 mol% DSPE-PEG3K in dichloromethane. Next, using a homogenizer (IKA, Ultra-Turrax^®^ T25 basic; Labortechnik, Staufen, Germany), the mixture was homogenized at 15,000 rpm for 2 min. The resultant emulsion was sonicated under instant cooling at 4 °C for 3 min at 45% power amplitude with 5 cycles using a probe-type ultrasonication (Sonoplus, HD 2070; Bandelin Electronics, Berlin, Germany). Finally, the organic solvent of emulsion was evaporated by magnetic stirring at 400 rpm for 3 h under negative pressure.

To prepare D-PRN and T-PRN, the abovementioned protocol was used, with the exception that either TRQ or DTX was not added, respectively. Accordingly, to investigate the cellular uptake behavior of PRN, both DTX and TRQ were replaced with DiI (red hydrophobic fluorescent probe) and C6 (green hydrophobic fluorescent probe), respectively. For further combined treatment, the D-PRN and T-PRN were physically mixed with a DTX:TRQ ratio of 1:1 (*w*/*w*) (D+T-PRN) to obtain the dual-carrier system. To observe the cellular uptake of PRN, DiI (a hydrophobic red fluorescent probe) and C6 (a hydrophobic green fluorescent probe) were loaded instead of DTX and TRQ, respectively. The empty-PRN was prepared without drugs or fluorescent probes. All prepared NLC samples were stored in a refrigerator at 4 °C and shielded from light and were used for subsequent experiments within 2 weeks of preparation. For in vivo intravenous injections, D-PRN, T-PRN, and D^T-PRN solutions were concentrated by ultracentrifugation at 14,000× *g* for 20 min at 25 °C using Amicon^®^ ultra-centrifugal filters (100 kDa MWCO; Millipore, Billerica, MA, USA). The D-PRN and D^T-PRN residues were diluted in 0.9% normal saline to obtain a final concentration of 6 mg/mL DTX. Moreover, the T-PRN residues were diluted to obtain a final concentration of 4 mg/mL TRQ.

### 3.6. Particle Size and ZP Analysis

A DLS particle size analyzer (Zetasizer Nano-ZS; Malvern Instruments, UK) was used to determine the average particle size, PDI, and ZP of the PRN samples. All measurements were performed in triplicate at 25 °C after 100-fold dilution of NLC samples in double-distilled water under the following conditions: light angle (173° backscattering), refractive index (1.330), cP viscosity (0.8872), and equilibration time (60 s).

### 3.7. TEM Analysis

To confirm the morphology of DTX- and/or TRQ-loaded NLC samples, TEM images were obtained using TEM (Talos L120C; FEI, Czech) at 120kV acceleration voltage. Briefly, a drop of the NLC samples was placed on a 200-mesh carbon film grid and negatively stained with 2% (*w*/*v*) phosphotungstic acid for 1 min. The grid was washed with deionized water, air-dried to evaporate the surface liquid at 25 °C, and observed using TEM.

### 3.8. High-Performance Liquid Chromatography (HPLC)

The amount of DTX or TRQ in the samples was quantified using HPLC (Waters^®^ Corporation, Milford, MA, USA) equipped with separating modules (Waters^®^ e2695) and a UV detector (Waters^®^ e2489) that was monitored by Empower^®^ 3 software for data processing. DTX was quantified using a C18 column (5 μm, 4.6 × 150 mm; Shiseido, Tokyo, Japan) with an isocratic mobile phase consisting of acetonitrile and water (55:45, *v*/*v*) at a flow rate of 1 mL/min at 25 °C. The sample volume was 50 μL, and the detection wavelength was 230 nm. For TRQ, the elution conditions involved gradient mobile phases, including solvent A (acetonitrile) and solvent B (0.05% trifluoroacetic acid). The flow rate was 1 mL/min, and the temperature was 30 °C. TRQ was detected at 254 nm, and the injection volume was 50 μL. The criteria of the gradient elution program were: from 0 to 8.0 min, 10–80% solvent A; from 8.0 to 13.0 min, 80% solvent A; from 13.0 to 13.1 min, 80–10% solvent A; and from 13.1 to 15.0 min, 10% solvent A.

### 3.9. Determination of EE and DL Capacity

Both the EE and DL of DTX and/or TRQ in various types of PRN samples were evaluated by the indirect method. Briefly, 500 μL drug-loaded PRN samples were placed into the 100 kDa MWCO Amicon^®^ ultra-centrifugal filters and centrifuged at 14,000× *g* and 25 °C for 20 min. The amount of free unencapsulated drugs in the filtrate was assayed by HPLC, as described earlier. The EE and DL were calculated as follows: EE (%) = (W_Total_−W_Free_)/W_Total_ × 100; DL (μg/mg) = (W_Total_−W_Free_)/W_Lipid_, where W_Total_, W_Free_, and W_Lipid_ represent the weight of the total drug added, the weight of free unencapsulated drug in the filtrate, and the weight of the total lipids added, respectively.

### 3.10. Assessment of Physical Stability of D^T-PRN

The physical stability of the D^T-PRN upon storage was investigated for particle size, ZP, and DL. Briefly, the D^T-PRN sample dispersed in distilled water was filled into glass vials with light shading and stored at 4 ± 1 °C for 4 weeks. Then, 1 mL of aliquots were withdrawn after 7, 14, 21, and 28 days of storage, and changes in particle size, ZP, and DL that occurred during storage were assessed in comparison to those on the day of production by using the above-described methods.

### 3.11. In Vitro Drug Release

The in vitro release assay of DTX or TRQ from DTX- and/or TRQ-loaded PRN samples was performed using the dialysis bag diffusion method [64]. Briefly, 1.5 mL solutions containing D-Sol + T-Sol, D+T-PRN, and D^T-PRN (DTX-equivalent amount of 300 μg) were added into dialysis bags with 300 kDa MWCO. The dialysis bags were tightly closed with clips and placed in 100 mL of PBS (0.01 M, pH 7.4) containing 1% (*w*/*v*) sodium dodecyl sulfate as a solubilizer, under continuous magnetic stirring at 100 rpm in the dark at 37 ± 0.5 °C. At the designated time intervals, 1 mL aliquots were drawn and replenished by adding an equal volume of fresh medium. The concentration of DTX or TRQ was determined by HPLC, as described above.

### 3.12. Cytotoxicity Assessment by WST-1

The cell viability of MCF7 or MCF7/ADR cells treated with the following formulations containing DTX and/or TRQ were evaluated by the WST-1 assay: D-Sol, D-PRN, T-Sol, T-PRN, D-Sol + T-Sol, D+T-PRN, and D^T-PRN. Briefly, cells were seeded into 96-well plates at a density of 1 × 10^4^ cells per well in 100 μL cell culture medium and incubated for 24 h. Following the cell attachment, the cells were incubated with culture medium containing DTX- and/or TRQ-loaded formulations at concentrations of 0.1, 1, 10, 100, 1000, and 10,000 ng/mL as the DTX- or TRQ-equivalent samples. After 24 h incubation, the drug-containing culture medium was discarded, and the cells were washed with PBS. Subsequently, 100 μL WST-1 solution (10%, *v*/*v*) was added, and the cells were incubated for 30 min at 37 °C. Finally, the absorbance of the formed formazan dye of each well was measured at 450 nm using a Flexstation 3 microplate reader (Molecular Devices LLC, Sunnyvale, CA, USA). Cell viability in the untreated group was considered as 100% viability. The relative viability of the treated cells was calculated as a percentage of the viability of the untreated control group. GraphPad Prism 7.05 (GraphPad Software Inc., San Diego, CA, USA) was used to calculate the IC_50_ via nonlinear regression.

### 3.13. Determination of CI and RE

For the evaluation of the combinational effects of DTX and TRQ, the CI was calculated using CompuSyn software ver. 1.0 (Biosoft, Cambridge, UK), based on the Chou–Talalay method [65]. The following equation was used: CI_x_ = (D_com_)_D_/(D_single_)_D_ + (D_com_)_T_/(D_single_)_T_, where (D_com_)_D_ and (D_com_)_T_ are the inhibitory concentrations (IC_x_) of DTX and TRQ in combination, respectively, and (D_single_)_D_ and (D_single_)_T_ are the IC_x_ values of DTX and TRQ as single agents, respectively. The CI values were plotted against the level of growth inhibition (Fa), which was calculated as follows: Fa = 1 − (percent viability of drug-treated cells/percent viability of untreated cells). A CI value of less than 1 represents a synergistic effect in the drug combination, whereas a value equal to 1 represents additive effects, and a value greater than 1 represents an antagonistic effect. RE was used to assess the degree of MDR reversal caused by treatment with drug-loaded PRN. The RE was calculated by the following formula [66]: RE = IC_50(D-Sol)_/IC_50(sample)_, where IC_50(D-Sol)_ is the IC_50_ value of DTX in cells treated with D-Sol, and IC_50(sample)_ is the IC_50_ value of DTX in cells treated with a DTX-loaded sample formulation.

### 3.14. Cell-cycle Analysis

The distribution in the cell-cycle phase was analyzed by the quantification of DNA contents using a PI and RNAse flow cytometry kit (Abcam, Cambridge, UK), in accordance with the manufacturer’s directions. Briefly, MCF7 and MCF7/ADR cells were seeded in 6-well plates at a density of 3 × 10^6^ cells/well, cultured for 24 h to allow attachment, and treated with DTX- and/or TRQ-loaded formulations to attain a final DTX-equivalent concentration of 10 ng/mL (TRQ-equivalent concentration of 10 ng/mL). After 24 h incubation, the cells were washed twice with cold PBS, detached with Accutase^®^ solution, and centrifuged at 500× *g* for 5 min. Then, the collected cells were fixed using cold 70% ethanol at 4 °C for 2 h. Subsequently, the cells were washed with pre-cooled PBS to remove residual ethanol, resuspended in 200 μL PBS, treated with RNase A (550 U/mL) and PI (0.05 μg/mL) in the dark for 30 min at 37 °C for staining, and then analyzed by flow cytometry with CellQuest Pro software (FACSCalibur; Becton Dickinson, Franklin Lakes, NJ, USA).

### 3.15. Cell Apoptosis Analysis

Apoptosis in the MCF7/ADR cells was observed using the Annexin V- FITC/PI apoptosis detection kit (BioLegend, San Diego, CA, USA), according to the manufacturer’s instructions. Briefly, cells were seeded at a density of 3 × 10^6^ cells/well into 6-well plates and cultured for 24 h. Then, the cells were incubated for 24 h with medium alone (untreated negative control) or medium containing test formulations (DTX- and/or TRQ-loaded formulations) at a final DTX-equivalent concentration of 10 ng/mL (TRQ-equivalent concentration of 10 ng/mL). Following the incubation, cells were washed twice with cold PBS, trypsinized, and collected by centrifugation at 500× *g* for 5 min. The collected cell pellets were reconstituted with 200 μL binding buffer containing 15 μL Annexin V-FITC and 5 μL PI, and the solution obtained was incubated at 25 °C for 20 min with light shading, in accordance with the manufacturer’s protocol. The percentage of apoptotic cells was measured by FACS analysis, and CellQuest Pro was used for data analysis. For each sample, 10,000 events were analyzed using the FL1 (green) and FL3 (red) channels for Annexin V-FITC and PI, respectively.

### 3.16. Cellular Uptake Assay by Receptor Blocking

To study the effect of Hpn blocking on PRN uptake, we performed a cellular uptake assay with ligand–receptor pre-binding using CLSM (Zeiss LSM 700; Carl Zeiss, Jena, Germany). Both Hpn-positive MCF7 and MCF7/ADR cells and Hpn-negative PC3 cells were seeded in a Lab-Tek II chamber slide with a cover (Thermo Fisher Scientific Nunc, Rochester, NY, USA) at a density of 1 × 10^5^ cells/well. After 24 h incubation, the medium was removed, washed twice with PBS, and pre-incubated with the culture medium containing empty-PRN (lipid concentration of 0, 6.7, 13.4, and 26.8 μg/mL) for 30 min. The cells were washed twice with PBS and treated with DiI-PRN (DiI-equivalent concentration of 50 ng/mL) for 30 min. Following incubation, the cells were washed twice with PBS and fixed with 4% formaldehyde in PBS for 15 min at 25 °C. The cells were mounted using Vectashield mounting medium with 4’,6-diamidino-2-phenylindole (DAPI; Vector Laboratories, Burlingame, CA, USA) to prevent fading and to ensure the staining of the nuclei.

### 3.17. Competitive Cellular Uptake Assay

The competitive cellular uptake behavior of the dual- and single-carrier systems was studied quantitatively using probe-laden PRN by determining the mean fluorescence intensity (MFI) of DiI and C6 with a flow cytometer. MCF7 and MCF7/ADR cells were seeded on a 12-well plate at a density of 2 × 10^5^ and allowed to adhere for 24 h. Thereafter, the medium was removed, and the cells were incubated for 30 min at 37 °C in a medium containing a physically mixed dual-carrier system of DiI-PRN and C6-PRN (DiI+C6-PRN) or DiI^C6-PRN (single-delivery system) (DiI concentration: 50 ng/mL; C6 concentration: 16.4 ng/mL). Cells incubated with only the medium were considered the untreated controls. After 30 min incubation, the cells were washed twice with PBS, detached from the wells using Accutase^®^ solution, centrifuged at 1000× *g* for 5 min for collection, and resuspended in 500 μL PBS for analysis using a flow cytometer. Data were analyzed using BD cell Quest Pro software. A total of 10,000 cells were analyzed for each determination using the FL1 and FL2 channels for C6 and DiI, respectively. Only viable cells were gated for analysis.

### 3.18. Evaluation of In Vivo Antitumor Efficacy

The in vivo antitumor activities were evaluated on the MCF7/ADR cells-bearing, female, BALB/c nude mice. To establish the MCF7/ADR tumor xenograft model, we subcutaneously inoculated female BALB/c athymic mice with a RPMI 1640 medium/Matrigel (100 μL, 50:50 *v*/*v*) suspension containing 1 × 10^7^ MCF7/ADR cells in the right flank. The tumor volume (mm^3^) was determined using digital calipers (Mitutoyo, Kawasaki, Japan) by measuring the tumor length (L) and width (W) and was calculated as: L × W^2^ × 0.5 [67]. Five weeks after inoculation, the tumor volume increased to 100–150 mm^3^, and the mice were randomly divided into four groups (*n* = 3): (1) normal saline (control), (2) D-PRN, (3) D+T-PRN (DTX:TRQ = 1:1, *w*/*w*), and (4) D^T-PRN (DTX:TRQ = 1:1, *w*/*w*). Each mouse was administered an intravenous injection via the tail vein at a dose of 5 mg DTX/kg, using a 21 G needle once a week for 3 weeks [68]. The first day of injection was designated as Day 0. The tumor volume and body weights were measured weekly for a total of 4 weeks. The percentage of TGI was calculated using the formula: TGI (%) = (1 – *V_t_/V_c_*) × 100, where *V_t_* is the tumor volume of the treated group, and *V_c_* is the tumor volume of the control group [69]. To detect potential adverse effects, general animal health, including food and water avoidance, impaired movement, and behavioral changes, were recorded. At the completion of the experiment, the mice were sacrificed by CO_2_ inhalation, and the tumors were excised and photographed.

### 3.19. Statistical Analysis

All data are presented as the mean ± standard deviation (SD). Data analysis was carried out with one-way independent-groups ANOVA, followed by Tukey’s test for post hoc comparison. For all analyses, differences were considered significant when *p*-values were less than 0.01 (*p* < 0.01), unless indicated otherwise.

## 4. Conclusions

Two types of co-delivery systems—D+T-PRN (dual-carrier type) and D^T-PRN (single-carrier type)—were developed with high DL capacity and an optimized synergistic weight ratio of DTX:TRQ (1:1). In both systems, TRQ exerted effective P-gp-inhibiting activity, which enhanced the intracellular accumulation of DTX and cell apoptosis. D^T-PRN exhibited a strong cytotoxic effect in both MCF7 (drug-sensitive) and MCF7/ADR (drug-resistant) cells, thereby demonstrating significantly increased MDR reversal efficiency. Competitive cellular uptake assays using fluorescent probes supported the excellent potential of single nanocarrier systems in avoiding receptor-binding competition. Furthermore, in an MCF7/ADR-xenograft mouse model, the D^T-PRNs enhanced the in vivo antitumor efficacy. Thus, simultaneous delivery of DTX and TRQ using co-encapsulated PRN has great potential for overcoming MDR in cancer. However, along with the dose adjustment, further in vivo evaluations of pharmacokinetics and biodistribution are still needed for the clinical application of these delivery systems.

## Data Availability

Data are contained within the article.

## Supplementary Materials

The following supporting information can be downloaded at: https://www.mdpi.com/article/10.3390/ph16030349/s1, Table S1a: Analysis of variance results of the apoptotic cell death; Table S1b: Post-hoc comparison results of the apoptotic cell death assay; Table S2: Analysis of variance results for the cellular uptake study; Table S3: Analysis of variance of the tumor growth inhibition assay.
