# Supplementary material for: Functionalized Lipid Nanocarriers for Simultaneous Delivery of Docetaxel and Tariquidar to Chemoresistant Cancer Cells
Source: Pharmaceuticals (Basel). 2023 Feb 24;16(3):349. doi: 10.3390/ph16030349 (PMC10058271; doi:10.3390/ph16030349)
Supplement: Supplementary file 1 [file pharmaceuticals-16-00349-s001.zip › pharmaceuticals-2228676-supplementary.pdf]

Table S1a. Analysis of variance results of the apoptotic cell death

Cases	Sum of squares	DF	Mean square	F-value	P-value
Treatment	1122.39	6	187.06	125.89	$p < 0.001$
Residual	20.80	14	1.49		

Table S1b. Post-hoc comparison results of the apoptotic cell death assay

		Mean difference	SE	t-value	P_{tukey}
D-PRN	D+T-PRN	–6.70	0.995	–6.74	< 0.001
	D^T-PRN	–11.08	0.995	–11.13	< 0.001
	D-Sol	1.80	0.995	1.81	0.565
	T-PRN	8.62	0.995	8.66	< 0.001
	T-Sol	8.48	0.995	8.52	< 0.001
	Untreated	8.36	0.995	8.40	< 0.001
D+T-PRN	D^T-PRN	–4.37	0.995	–4.39	0.008
	D-Sol	8.50	0.995	8.54	< 0.001
	T-PRN	15.32	0.995	15.39	< 0.001
	T-Sol	15.18	0.995	15.26	< 0.001
	Untreated	15.07	0.995	15.14	< 0.001
D^T-PRN	D-Sol	12.87	0.995	12.93	< 0.001

	T-PRN	19.69	0.995	19.79	< 0.001
	T-Sol	19.56	0.995	19.65	< 0.001
	Untreated	19.44	0.995	19.53	< 0.001
D-Sol	T-PRN	6.82	0.995	6.85	< 0.001
	D-Sol + T-Sol	6.68	0.995	6.72	< 0.001
	T-Sol	6.57	0.995	6.60	< 0.001
T-PRN	T-Sol	–0.14	0.995	–0.14	> 0.999
	Untreated	–0.25	0.995	–0.26	> 0.999
T-Sol	Untreated	–0.12	0.995	–0.12	> 0.999

Table S2. Analysis of variance results for the cellular uptake study

Cell	Probe	Cases	Sum of squares	DF	Mean square	F-value	P-value
MCF7	C6	Treatment	2484.09	2	1242.05	218.62	$p < 0.001$
		Residual	34.09	6	5.68		
	DiI	Treatment	402.79	2	201.39	235.11	$p < 0.001$
		Residual	5.14	6	0.86		
MCF7/ADR	C6	Treatment	933.93	2	466.97	1011.89	$p < 0.001$
		Residual	2.77	6	0.461		
	DiI	Treatment	165.18	2	82.59	821.96	$p < 0.001$
		Residual	0.60	6	0.1		

Table S3. Analysis of variance of the tumor growth inhibition assay

Cases	Sum of squares	DF	Mean square	F-value	P-value
Treatment	2457.00	2	1229.00	53.64	$p < 0.001$
Residual	137.40	6	22.91		
